# Irisin prevents trabecular bone damage and tumor invasion in a mouse model of multiple myeloma

**DOI:** 10.1093/jbmrpl/ziae066

**Published:** 2024-05-23

**Authors:** Roberta Zerlotin, Angela Oranger, Patrizia Pignataro, Manuela Dicarlo, Lorenzo Sanesi, Clelia Suriano, Giuseppina Storlino, Rita Rizzi, Anna Mestice, Sante Di Gioia, Giorgio Mori, Maria Grano, Graziana Colaianni, Silvia Colucci

**Affiliations:** Department of Precision and Regenerative Medicine and Ionian Area, University of Bari, 70124 Bari, Italy; Department of Precision and Regenerative Medicine and Ionian Area, University of Bari, 70124 Bari, Italy; Department of Translational Biomedicine and Neuroscience, University of Bari, 70124 Bari, Italy; Department of Precision and Regenerative Medicine and Ionian Area, University of Bari, 70124 Bari, Italy; Department of Clinical and Experimental Medicine, University of Foggia, 71122 Foggia, Italy; Department of Precision and Regenerative Medicine and Ionian Area, University of Bari, 70124 Bari, Italy; Department of Clinical and Experimental Medicine, University of Foggia, 71122 Foggia, Italy; Department of Precision and Regenerative Medicine and Ionian Area, University of Bari, 70124 Bari, Italy; Department of Precision and Regenerative Medicine and Ionian Area, University of Bari, 70124 Bari, Italy; Department of Clinical and Experimental Medicine, University of Foggia, 71122 Foggia, Italy; Department of Clinical and Experimental Medicine, University of Foggia, 71122 Foggia, Italy; Department of Precision and Regenerative Medicine and Ionian Area, University of Bari, 70124 Bari, Italy; Department of Precision and Regenerative Medicine and Ionian Area, University of Bari, 70124 Bari, Italy; Department of Translational Biomedicine and Neuroscience, University of Bari, 70124 Bari, Italy

**Keywords:** irisin, multiple myeloma, bone damage, sclerostin, RankL, Opg

## Abstract

Bone disease associated with multiple myeloma (MM) is characterized by osteolytic lesions and pathological fractures, which remain a therapeutic priority despite new drugs improving MM patient survival. Antiresorptive molecules represent the main option for the treatment of MM-associated bone disease (MMBD), whereas osteoanabolic molecules are under investigation. Among these latter, we here focused on the myokine irisin, which is able to enhance bone mass in healthy mice, prevent bone loss in osteoporotic mouse models, and accelerate fracture healing in mice. Therefore, we investigated irisin effect on MMBD in a mouse model of MM induced by intratibial injection of myeloma cells followed by weekly administration of 100 μg/kg of recombinant irisin for 5 wk. By micro-Ct analysis, we demonstrated that irisin improves MM-induced trabecular bone damage by partially preventing the reduction of femur Trabecular Bone Volume/Total Volume (*P* = .0028), Trabecular Number (*P* = .0076), Trabecular Fractal Dimension (*P* = .0044), and increasing Trabecular Separation (*P* = .0003) in MM mice. In cortical bone, irisin downregulates the expression of Sclerostin, a bone formation inhibitor, and RankL, a pro-osteoclastogenic molecule, while in BM it upregulates Opg, an anti-osteoclastogenic cytokine. We found that in the BM tibia of irisin-treated MM mice, the percentage of MM cells displays a reduction trend, while in the femur it decreases significantly. This is in line with the in vitro reduction of myeloma cell viability after 48 h of irisin stimulation at both 200 and 500 ng/mL and, after 72 h already at 100 ng/mL rec-irisin. These results could be due to irisin ability to downregulate the expression of Notch 3, which is important for cell-to-cell communication in the tumor niche, and Cyclin D1, supporting an inhibitory effect of irisin on MM cell proliferation. Overall, our findings suggest that irisin could be a new promising strategy to counteract MMBD and tumor burden in one shot.

## Graphical Abstract

**Graphical Abstract:**
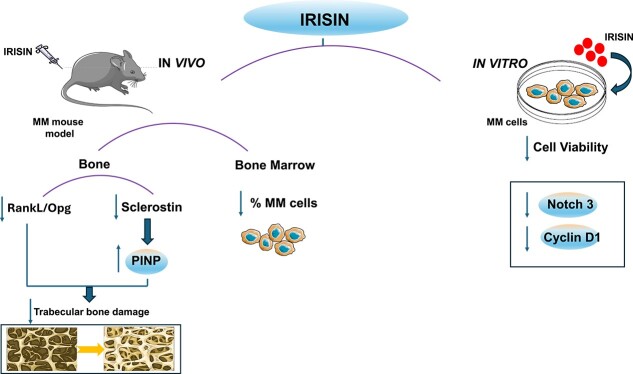

We thank Servier Medical Art (https://smart.servier.com/) for providing free image software to build the figure.

## Introduction

Bone pain and fractures are the first symptoms and evidence that lead to the diagnosis of multiple myeloma (MM), which begins in the BM with the proliferation of a malignant plasma cell clone. These cells find in the BM an ideal milieu for both their proliferation and the release of molecules able to alter bone cell activities, resulting in bone resorption excess and matrix neo-deposition decrease. This derangement is responsible for osteolytic lesions, a hallmark of MM-associated bone disease (MMBD),^1^ contributing to making bones thinner, weaker, and prone to pathological fractures.^2^

Due to skeletal fragility, associated fracture risks, and, in parallel, lower muscle strength,^3^ physical activity, although beneficial in various pathological conditions and cancer types, has not been recommended for a long time to MM patients. However, this claim has recently been overturned, as some evidence suggested that these patients can undergo an individual program of exercise with positive effects on quality of life, fatigue, pain, mobility, sleep, and mood.^4^ From a pharmacological point of view, despite advances that have been made in MM management and generated prolonged survival, MMBD treatment is still an open challenge. To date, the use of antiresorptive drugs, bisphosphonates, and denosumab is the main option for the treatment of MMBD,^5^ but osteoanabolic molecules, such as anti-Dickkopf-related protein 1 (anti-Dkk1) and anti-Sclerostin, as well as the identification of new therapeutic strategies, are currently under investigation.^6^

Therefore, this paper is focused on the effect of irisin, the new bone-anabolic exercise-mimetic hormone, on MMBD. Following the discovery of irisin,^7^ we demonstrated the molecule’s ability to enhance bone mass in healthy mice^8^ and to prevent and restore bone loss and muscle atrophy in a disuse-induced osteosarcopenic mouse model.^9^ Irisin directly targets the bone-forming cells, osteoblasts, and the mechanosensor and master regulators of bone remodeling, osteocytes. The myokine promotes the differentiation and activity of osteoblasts,^10^ and the viability of osteocytes in vivo and in vitro^11^ exerting its effect on bone through a receptor mechanism mediated by αVβ3 integrin.^12^ The anabolic role of irisin also emerged from data showing that the molecule improves bone microarchitecture in a mouse model of androgen-deficiency-induced osteoporosis, and protects ovariectomized mice and rats from bone loss.^13^^,^^14^ In line with its beneficial effect on bone, irisin accelerates the fracture healing process by favoring osteogenesis and angiogenesis in mice.^15-17^

To date, human studies have been observational only and have shown a positive correlation of irisin serum levels with both vertebral and femoral BMD in elderly subjects.^18^ Furthermore, circulating irisin levels have been shown to increase in the fracture union process,^19^ and negatively associated with vertebral fragility fractures in post-menopausal women.^20^ Recently, studies on the effect of irisin on tumors have received extensive attention. They have demonstrated lower expression of the irisin precursor in different cancer tissues (breast and gastric),^21^^,^^22^ as well as in osteosarcoma,^23^ one of the most common primary tumors of the bone. In addition, in vitro irisin stimulation, although at different concentrations and times of treatment, induces the inhibition of cancer cell proliferation,^23-28^ suggesting an anti-cancer role of the myokine. However, the effect of irisin on MMBD and malignant plasma cells has not been studied so far, and we sought to investigate its possible involvement in this study. Moreover, based on the recently emerged positive effect of individualized exercise in MM patients, we explored the hypothesis that irisin, acting as an exercise-mimetic molecule, could be the molecular linker of these positive outcomes in MM. In agreement with our hypothesis, recent data have shown that after exercise, patients affected by other diseases showed increased serum irisin levels and improvement in depression and fatigue.^29^ These findings are further supported by data obtained in young mice showing the effects of irisin in reducing depression and anxiety,^30^^,^^31^ suggesting that irisin could be a viable molecular candidate in influencing mood in MM patients and paving the way for further studies in this field.

Therefore, here we investigated the effect of irisin on bone damage, malignant cell BM invasion, and behavioral parameters related to locomotor activity in an MM-induced mouse model, as well as on plasma cell proliferation in vitro. Our in vivo and in vitro findings suggest that irisin improves MMBD and at the same time inhibits malignant cell growth.

## Materials and methods

### Myeloma cell line and culture condition

Murine 5TGM1-enhanced green fluorescent protein (5TGM1-eGFP) cell line (kindly provided by Katherine Weilbaecher, Washington University School of Medicine in St. Louis, Department of Medicine, Division of Oncology) was cultured in Iscove’s Modified Dulcecco’s Medium (IMDM, 12440-053, gGibco, Thermo Fisher Scientific), complemented with 10% FBS (A5256801, Gibco, Thermo Fisher Scientific) and 1% streptomycin/ penicillin (15140122, Gibco, Thermo Fisher Scientific), at 37 °C in a 5% CO_2_ humid atmosphere.

### Study design

All experimental protocols were carried out in compliance with the European Law Implementation of Directive 2010/63/EU. Experimental procedures were examined and approved by the Veterinary Department of the Italian Ministry of Health (Project: 12-2022-PR). Nine-week-old immune-competent C57BKALwRijHsd (ENVIGO, *n* = 24) female mice were randomly divided into 3 groups: Sham-vehicle mice (Sham-vehicle), injected intratibially with vehicle (physiological solution sterilized by 0.22 m filtration) and 2 groups of mice injected intratibially with 10^5^ murine 5TGM1-eGFP cells and treated intraperitoneally with vehicle (MM-vehicle) or with 100 μg/kg recombinant (rec)-irisin (MM-irisin) once/wk for 5 wk. We performed the study on female mice, as reported in other studies.^32^ All experiments on mice were performed in the same cohort of mice.

### Procedure for intratibial injection

The mice were anesthetized in accordance with the UCSF guidelines for rodent anesthesia with xylazine (10 mg/kg) (1 720 407, Sigma-Aldrich, Merck) and ketamine (100 mg/kg) (BP736, Sigma-Aldrich, Merck); the operator used sterile survival procedures according to the IACUC guidelines. C57BKALwRijHsd female mice were bilaterally injected intratibially with 10^5^ murine 5TGM1-eGFP MM cells^33^ or saline, and then the animals were housed separately and monitored closely for about 3 hours (h) until they fully recovered from anesthesia. At the end of the recovery period (24 h after surgery), the animals were housed, 3-4 animals per cage, with a 12-h light/dark cycle, in an environment with controlled temperature and with regular access to water and food (Harlan Teklad 2019; SDS Special Diets Services).

### Recombinant irisin treatment

**Figure 1:**
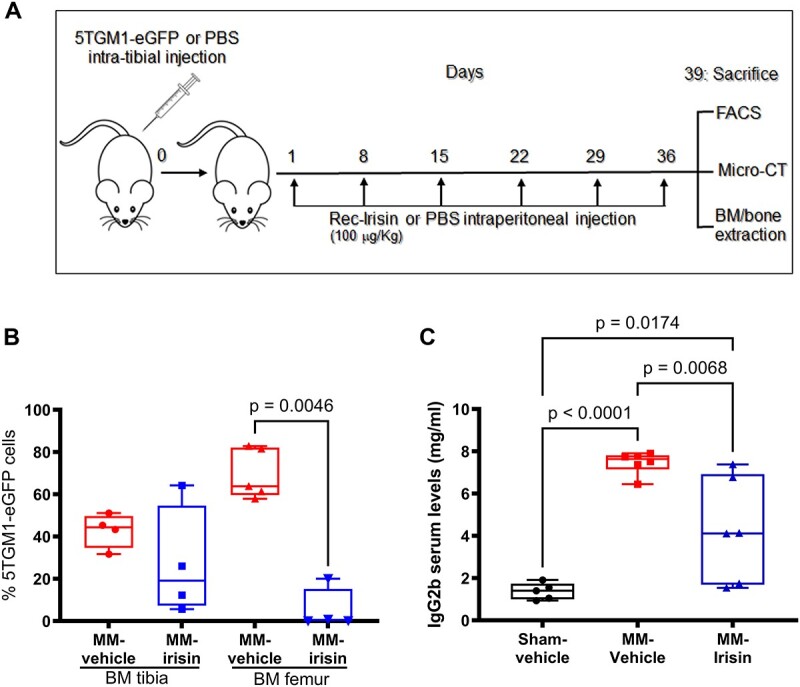
Irisin reduces femoral BM malignant cell invasion and serum levels of IgG2b after intratibial injection in mice. Experimental design (A). Percentage of 5TGM1-eGFP cells detected within the BM from the tibia and femur of 5TGM1-eGFP-bearing female mice treated for 5 wk with vehicle (MM-vehicle) or 100 μg/kg rec-irisin (MM-irisin) (B). IgG2b serum levels in Sham-vehicle (*n* = 5), MM-vehicle (*n* = 6), and MM-irisin mice (*n* = 6) (C). Kruskal–Wallis test or one-way ANOVA with Tukey’s multiple comparisons tests was performed. Data are presented as a box-and-whisker plot with median and interquartile ranges, from max to min, with all data points shown.

One day after 5TGM1-eGFP myeloma cells or saline intratibial injection, mice that survived in good health were intraperitoneally (i.p.) injected once a week for 5 wk with either the vehicle (sterile physiologic solution) or with 100 μg/kg rec-irisin (AG40B-0128-C010, Adipogen International, San Diego). Lyophilized irisin containing sodium phosphate buffer was dissolved in sterile distilled water, following the manufacturer’s instructions. This frequency and amount of treatment were selected because they successfully induced bone increase in other mouse models such as osteoporotic ones.^9^^,^^11^ The group of Sham-vehicle mice (*n* = 6) and MM-vehicle mice (*n* = 6) was injected with the vehicle, while the group of MM-irisin mice (*n* = 6) with 100 μg/kg rec-irisin once a week for 6 times (Figure 1A).

### Behavioral assessment

To evaluate the well-being and health of mice, all behavioral changes were noted each day, like the posture and facial features, that is, ear and whisker position, orbital tightening, and nose/cheek bulge, using the Mouse Grimace Scale.^34^ Moreover, to assess the locomotor abilities, the Open Field Test (OFT) was carried out by a trained researcher. Additional behavioral paradigms were not performed to avoid further stressing the mouse.

Concerning the OFT, animals were conducted into the testing room 30 min before starting the experiment. The OFT was performed on 35th d after the injection of myeloma cells, that is, when MM-mice developed the first behavioral signs of the disease (motor slowing, decreased exploration, pain). The OFT consisted of a polycarbonate, transparent cage with bedding on the bottom, similar to their home cage but slightly bigger (37 × 21 × 18 cm). Mice were positioned individually in the center of apparatus and enabled to explore the environment for 10 min. Their locomotion was recorded to evaluate the total distance moved, velocity, number of rearing (vertical exploration), and digging (exploratory behavior, index of protection, and well-being) for analyses.

### Animal sacrifice and tissue harvesting

At day 39, when MM-mice blatantly showed physical signs of discomfort (ie, lameness or swelling of 1 or more limbs), all mice were euthanized with CO_2_, and then their tissues were removed surgically. The right femurs were dissected, fixed with 4% (v/v) paraformaldehyde (158 127, Sigma-Aldrich, Merck) at 4 °C for 18 hs, and examined for microarchitecture analysis. The left femurs were subjected to flushing to separate BM from bone segments. The latter were lysed for western blot analysis, while BMs collected after flushing were subjected to FACS and real-time PCR evaluations (Figure 1A). Standard biosecurity and institutional safety protocols were followed during the execution of the experimental procedures. Power analysis: for *P* < .05; *n* = 4–6 mice/group. Sample sizes were chosen based on pilot studies and prior related work.

### Micro-CT analysis

MicroCT (μCT) scanning was performed to measure the morphological indices of the femurs’ metaphyseal regions. Femurs were placed in 70% ethanol and introduced into the specimens while rotating around their long axes in line with the scanner’s vertical axis. Bruker Skyscan 1172 version 1.5 (Bruker) was used to acquire images. Specific parameters were utilized: peak tube potential = 59 kV; voxel size 6 μm^3^; X-ray intensity = 167 μA; 0.5 mm aluminum foil; frame averaging = 3; sample rotation = 360°; rotation step = 0.4°; exposure time = 1185 ms. SkyScan reconstruction software (NRecon version 1.6.10.1) (Bruker) was used to reconstruct raw images. Subsequently, 3-dimensional cross-sectional image data sets were produced using a 3-dimensional cone-beam algorithm. We utilized the following setup: unified attenuation (output) range = 0.01–0.15; obtained data were corrected for possible mismatches of overlapping sub-scans; mild beam-hardening correction = 40%; ring artifact correction = 5. The acquired images that were utilized in the following phase were saved in 8-bit PNG format. To compute volumetric BMD and TMD and for calibration, a set of 3 hydroxyapatite (HA) phantoms (0.25 and 0.75 g·cm^−3^, and 2 mm diameter) was scanned using the same setup. By utilizing Skyscan CT Analyzer (CTAn version 1.20.8.0) software (Bruker, Kontich, Belgium), we calculated structural indices on reconstructed images. Trabecular and cortical bones were divided using a custom processing algorithm in CTAn, depending on the different structure thicknesses. Evaluation of cortical bone was done at the femoral midshaft. The midpoint of the bone, which is for all mice equal to half the distance between the proximal point of the femoral head and the distal condyle, is the center of the scan volume of the femoral midshaft. The analysis of the cortical bone was performed by using a region of 150 slices, starting 9 mm distal to the metaphysis. Trabecular bone was evaluated in the proximal metaphysis region, beginning proximal to the distal growth plate and moving distally for 200 slices. The trabecular parameters evaluated comprise bone volume fraction (Tb. BV/TV), Trabecular Number (Tb. Number), Trabecular Fractal Dimension (Tb. FD), Trabecular Separation (Tb. Separation), Trabecular Thickness (Tb. Thickness), BMD, Trabecular Open Porosity (Tb. Open Po), and Trabecular Degree of Anisotropy (Tb. DA). The cortical parameters included Tissue Mineral Density (Ct. TMD), Cortical Mineral Density (Ct. BMD), Cortical Thickness (Ct. Thickness), and Cortical polar Moment of Inertia (Ct. pMOI). We followed the guidelines of Bouxsein et al.^35^ for all acquisition methods, image analyses, and terminology parameters analyzed.

### MTT assay

The effect of irisin on murine 5TGM1 cell viability was evaluated using the 3-(4,5-dimethylthiazol-2-yl)-2,5-diphenyltetrazolium bromide (MTT) assay. Myeloma cells were plated at 100 000 cells per well in 96-well tissue-culture plates and were cultured with IMDM supplemented with 10% FBS and 1% penicillin/streptomycin. These cells were immediately stimulated with 0, 100, 200, or 500 ng/mL rec-irisin for different time points (24, 48, and 72 hs). In another experiment, 5TGM1 cells were pre-treated or not for 10 min with 20 nM of CycloRGDyK (Selleckchem, N. S7844) or DMSO (the same volume present in 20 nM of CycloRGDy) and then stimulated with 0 or 200 ng/mL rec-irisin for 72 hs. Subsequently, 1.2 mmol/L MTT (Sigma Aldrich) was added to the culture media and then incubated in a humidified 5% CO_2_ atmosphere at 37 °C; the reaction ended by adding 150 μL 0.04 N HCl in absolute isopropanol after 4 hs, followed by reading the optical density at 570 nm by means of an automatic plate reader (550 Microplate Reader; Bio-Rad Laboratories). The percentage of cell viability was calculated compared to 24 hs 0 ng/mL rec-irisin group (Figure 6A) or to 72 hs untreated group (Figure 6B).

### Real-time PCR

BM was obtained by flushing from femurs from Sham-vehicle, MM-vehicle, and MM-irisin mice; then RankL, Opg, and Gapdh were evaluated by Real-Time PCR. 5TGM1 murine cell line, treated or not for 8 hs with 100 and 200 ng/mL rec-irisin, was subjected to Notch 1, Notch 2, Notch 3, Notch 4, Jagged 1, Jagged 2, Cyclin D1, and Gapdh evaluation by Real-Time PCR. Total RNA from femur BM or from differently treated 5TGM1 cells was obtained using spin columns (Qiagen, Hilden, Germany). By using iScript Reverse Transcription Supermix (Bio-Rad Laboratories), reverse transcription was performed in the thermocycler (My cycler; Bio-Rad Laboratories) and Real-time PCR by using SsoFast EvaGreen Supermix (Bio-Rad Laboratories, Hercules) on the CFX96 real-time system (Bio-Rad Laboratories) for 40 cycles (denaturation 95 °C for 5 s; annealing/extension 60 °C for 10 s), after activating the enzyme with a 30 s step at 95 °C. The Primer Blast (https://www.ncbi.nlm.nih.gov/tools/primer-blast/) was used to design the primers that span an exon–exon junction. Oligonucleotide sequences for target genes are reported in Primers Supplemental Table. Each transcript was examined in triplicate, and quantitative measures were evaluated using the ∆∆CT method and were expressed as a relative fold change from control.

### Western blot analysis

Left femurs, depleted from BM from Sham-vehicle, MM-Vehicle, and MM-irisin of mice, were homogenized by using Ultra-Turrax T8 (Ika, Staufen im Breisgau, Germany) and lysed to obtain a protein extract. About 30 μg of protein were subjected to western blot analysis to detect RankL, Opg, Sclerostin, Dkk1, and β-actin protein levels. Each lysate was subjected to sodium dodecyl sulfate-polyacrylamide gel electrophoresis gel and then transferred to nitrocellulose membranes (Hybond; Amersham Pharmacia). The blots were incubated at 4 °C overnight with the anti-RankL, anti-Opg, anti-Sclerostin, anti-Dkk1, and anti-β-actin primary Ab (Antibodies Supplemental Table). Then, the blots were incubated with the specific fluorescent-dye-conjugated secondary Ab (Antibodies Supplemental Table), and finally, the LI-COR’s Odyssey Infrared Imaging System (LI-COR Biotechnology Lincoln) was used to reveal specific reactions.

### Enzyme-linked immunosorbent assay

Murine IgG2b circulating levels were determined using a specific mouse IgG2b ELISA Kit (Antibodies, A1570). The quantification of circulating levels of N-terminal propeptide of type I procollagen and C-terminal telopeptides of type I collagen (CTX) was performed using mouse PINP ELISA Kit (Antibodies, A78633) and mouse CTX-I ELISA kits (Antibodies, ABIN6963046), respectively.

### Statistical analysis

Shapiro–Wilk normality test was used to evaluate sample distribution. Parameters were expressed as the median and IQR using GraphPad Prism 9.5 (GraphPad Software, Inc.). For normal distributed values, we performed a ANOVA with Tukey’s multiple comparisons tests, whereas for non-normal distributed values, we utilized Mann–Whitney test or a Kruskal–Wallis’ multiple comparison test. All data are shown as box-and-whisker plots with median and interquartile ranges, from max to min, with all data points shown. For *P* < .05, differences were considered significant.

## Results

### Irisin reduces femoral BM malignant cell invasion and circulating IgG2b levels in MM mice

To study the effect of irisin on MMBD, we used an MM-induced mouse model obtained by intratibial 5TGM1-eGFP cells injection.^33^ We divided the mice into 3 groups: the first group was intratibially injected with PBS (Sham-vehicle), while the second and the third with 5TGM1-eGFP and then treated with PBS (MM-vehicle) or irisin (MM-irisin) respectively, once a week for 39 d (Figure 1A). The success of the MM establishment and invasion was evaluated by collecting the BM from the tibia and femur of the mice and performing flow cytometry analysis. We demonstrated the onset of MM in both vehicle- and irisin-treated mice groups, with 45% (median value) and 18% (median value) positivity of GFP + 5TGM1 cells in the injected site (tibia), respectively (Figure 1B). Although the percentage of positivity showed a reduction trend in the BM of MM-irisin-treated mice compared with untreated ones, unexpectedly, there were no significant differences between them due to the high variability of the results. We also assessed the invasion of MM cells into the femur of vehicle mice by demonstrating that the percentage of GFP + 5TGM1 cells was not significantly different from that detected in the tibia of the same mice, indicating homing of MM cells from the tibia to the femur (Figure 1B). Similarly, the percentage of GFP + 5TGM1 cells in the femur of irisin-treated mice was not statistically different from that in the tibia (Figure 1B). Importantly, the percentage of GFP + 5TGM1 cells within femur BM was significantly reduced (*P* = .0046) in irisin-treated mice (median value: 2%) compared with vehicle once (median value: 64%) (Figure 1B), suggesting that irisin treatment takes part in the reduction of tumor burden. To further support the success of the MM establishment and irisin effect on tumor burden, we also determined IgG2b levels in the sera of Sham-vehicle, MM-vehicle, and MM-irisin mice. As shown in Figure 1C, IgG2b serum levels were 5-fold higher (*P* < .0001) in the MM-vehicle group (median value: 1.402 mg/mL) compared with Sham-vehicle one (median value: 7.633 mg/mL); irisin treatment significantly reduced (*P* = .0068) IgG2b serum levels compared with vehicle one (median value: 4.116 mg/mL), without reaching the levels of Sham-vehicle mice values (*P* = .0174 Sham-vehicle vs MM-irisin). This result further supports irisin involvement in reducing tumor burden.

### Irisin partially prevents trabecular bone microarchitecture alteration in the femur of MM-mice

**Figure 2:**
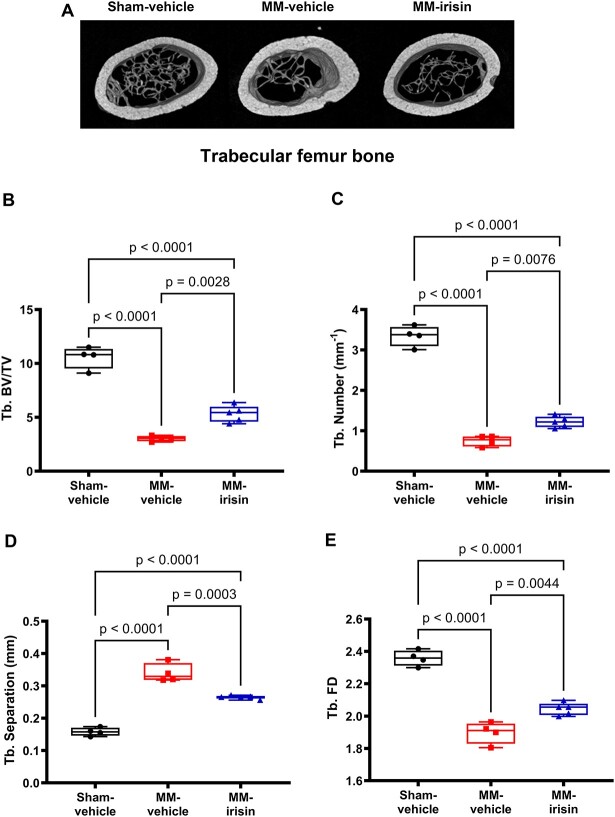
Irisin prevents trabecular bone loss in the femur of MM mice. Representative micro-CT-generated section images (A) and calculated trabecular parameters (B) of femur harvested from Sham-vehicle mice, MM-vehicle mice, and MM-irisin mice after 5 wk of treatment. Trabecular bone parameters included Trabecular Bone Volume/Total Volume (BV/TV), Trabecular Number (Tb. Number); Trabecular Separation (Tb. Separation), and Trabecular Fractal Dimension (Tb. FD) evaluated on Sham-vehicle (*n* = 4), MM-vehicle (*n* = 4), and MM-irisin (*n* = 5) mice (B, C, D, E). A one-way ANOVA with Tukey’s multiple comparisons tests was performed. Data are presented as box-and-whisker plots with median and interquartile ranges, from max to min, with all data points shown.

To evaluate whether irisin protects against MM-associated bone decrease, we performed bone histomorphometry analysis of the femur of Sham (*n* = 4), MM-vehicle (*n* = 4), and MM-irisin (*n* = 5) female mice. Specifically, we evaluated changes in the structure of bone by micro-CT analysis on both trabecular and cortical bone. Qualitative observations of micro-CT-generated section images of femurs highlighted trabecular bone loss in MM-vehicle mice compared to Sham mice, with a partial rescue of trabecular architecture in MM-irisin-treated ones (Figure 2A). Quantitative evaluation showed a significant reduction in Trabecular BV/TV (−3.48-fold, *P* < .0001, Figure 2B), Trabecular Number (Tb. Number: −4.46-fold, *P* < .0001, Figure 2C), and Trabecular Fractal Dimension (Tb. FD: −1.24-fold, *P* < .0001, Figure 2E), and a significant increase of Trabecular Separation (Tb. Separation: +2.15-fold, *P* < .0001, Figure 2D) in the femur of MM mice compared with Sham mice. Interestingly, irisin treatment, although partially (*P* < .0001, Sham vehicle vs MM irisin), significantly prevented the reduction of trabecular BV/TV (*P* = .0028), Tb. Number (*P* = .0076), and Tb. FD (*P* = .0044) (Figure 2B, C, and E) and the increase of Tb. Separation (*P* = .0003) (Figure 2D**)**. Differences were not found between MM-vehicle and -irisin mice for Tb. Open Po, Tb. DA, Tb. BMD, and Tb. Thickness (data not shown). In addition, cortical bone parameters were not affected by irisin administration (data not shown).

### Irisin modulates RankL, Opg, and Sclerostin expression

Cytokines involved in bone remodeling contribute to the onset of MMBD,^36-39^ and given that irisin is able to modulate some of them,^9^ we evaluated gene expression of RankL and Opg, as well as the protein levels of RankL, Opg, Sclerostin and Dkk1 in Sham mice and in our vehicle- and irisin-treated MM-induced mice.

**Figure 3:**
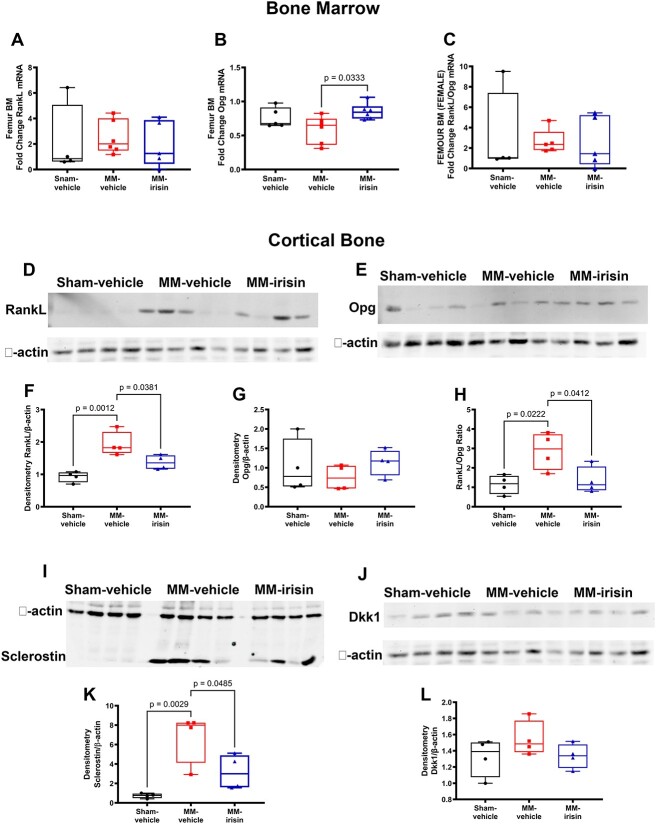
Irisin modulates RankL, Opg, and Sclerostin expression in MM mice. Quantitative PCR (qPCR) showing modulation of mRNA expression levels of RankL (A), Opg (B), and RankL/Opg ratio (C) assayed on the femoral BM of Sham-vehicle mice (*n* = 4 or *n* = 5), MM-vehicle mice (*n* = 5 or *n* = 6) and MM-irisin mice (*n* = 5 or *n* = 6) after 5 wk of treatment. Gene expression was normalized to Gapdh and plotted as a fold increase from the Sham-vehicle group of mice. Kruskal–Wallis test (RankL and RankL/Opg ratio) or one-way ANOVA with Tukey’s multiple comparisons tests (Opg) were performed. Data are presented as box-and-whisker plots with median and interquartile ranges, from max to min, with all data points shown. Western immunoblotting (D, E, I, J) and densitometric analysis **(**F, G, K, L) showing RankL, Opg, Sclerostin, and Dkk1 expression normalized to β-actin in the femur of Sham-vehicle mice (*n* = 4), MM-vehicle mice (*n* = 4), and MM-irisin mice (*n* = 4) after 5 wk of treatment. RankL/Opg ratio of densitometric analysis (H). A one-way ANOVA with Tukey’s multiple comparisons tests was performed. Data are presented as box-and-whisker plots with median and interquartile ranges, from max to min, with all data points shown.

Interestingly, by using RT-PCR, we found that irisin significantly increased Opg mRNA expression in the BM of femurs from MM mice (*n* = 6) compared to MM vehicle mice (*n* = 6) (*P* = .0333, Figure 3B), without any modulation of RankL levels (Figure 3A) and RankL/Opg ratio (Figure 3C). We also evaluated protein levels of RankL (Figure 3D) and Opg (Figure 3E) in the cortical bone of femurs from Sham-vehicle (*n* = 4), MM-vehicle (*n* = 4), and MM-irisin (*n* = 4) mice. By densitometric analysis, we demonstrated the increase of RankL in the MM-vehicle group compared with Sham-vehicle one (*P* = .0012), and its significant reduction in irisin-treated mice (*P* = .0381, MM-vehicle vs MM-irisin) (Figure 3F). Although Opg protein levels were not modulated in the 3 groups (Figure 3G), RankL/Opg ratio was significantly increased in the MM-vehicle group compared with Sham-vehicle one (*P* = .0222) and downregulated after irisin treatment (*P* = .0412, MM-vehicle vs MM-irisin) returning to Sham-vehicle levels (Figure 3H). These results indicate for the first time that irisin decreases RankL and RankL/Opg ratio in cortical bones of MM mice.

Furthermore, we analyzed protein levels of Sclerostin (Figure 3I) and Dkk1 (Figure 3J) in the cortical bone of femurs from Sham (*n* = 4), MM-vehicle (*n* = 4), and MM-irisin (*n* = 4) mice. Densitometric analysis showed that irisin treatment significantly reduced Sclerostin protein expression (*P* = .0485), which was higher in MM-vehicle mice compared to Sham ones (*P* = .0029) (Figure 3K). Differently, the expression levels of Dkk1 were not significantly modulated in MM (Figure 3L).

### Irisin increases PINP serum levels in MM mice

**Figure 4:**
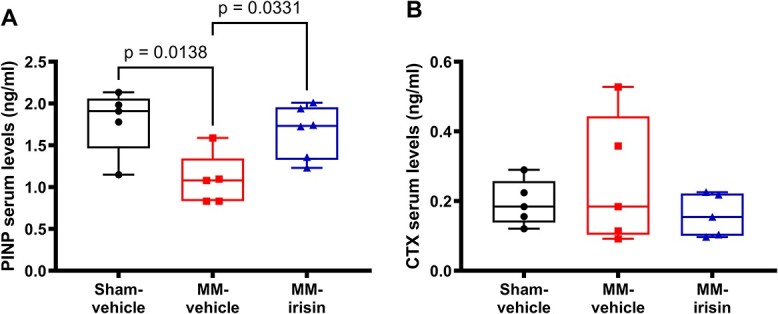
Irisin increases PINP serum levels in MM mice. N-terminal propeptide of type I procollagen (PINP) (A), and C-terminal telopeptides of type I collagen (CTX), (B) in Sham (*n* = 5), MM-vehicle (*n* = 5), and MM-irisin (*n* = 5 or *n* = 6) mice. A one-way ANOVA with Tukey’s multiple comparisons tests was performed. Data are presented as box-and-whisker with median and interquartile ranges, from max to min, with all data points shown.

To further investigate irisin involvement in MMBD, we quantified serum markers of bone formation (PINP) and bone resorption (CTX) in Sham (*n* = 5), MM-vehicle (*n* = 5), and MM-irisin (*n* = 6 or *n* = 5) groups of mice. Despite no significant differences were obtained from CTX quantification (Figure 4B), irisin administration promotes a significant increase of PINP serum levels in MM mice (*P* = .0331) that were lower in MM-vehicle mice compared to Sham ones (*P* = .0138, Figure 4A).

### Irisin improves MM mice explorative abilities in the OFT

**Figure 5:**
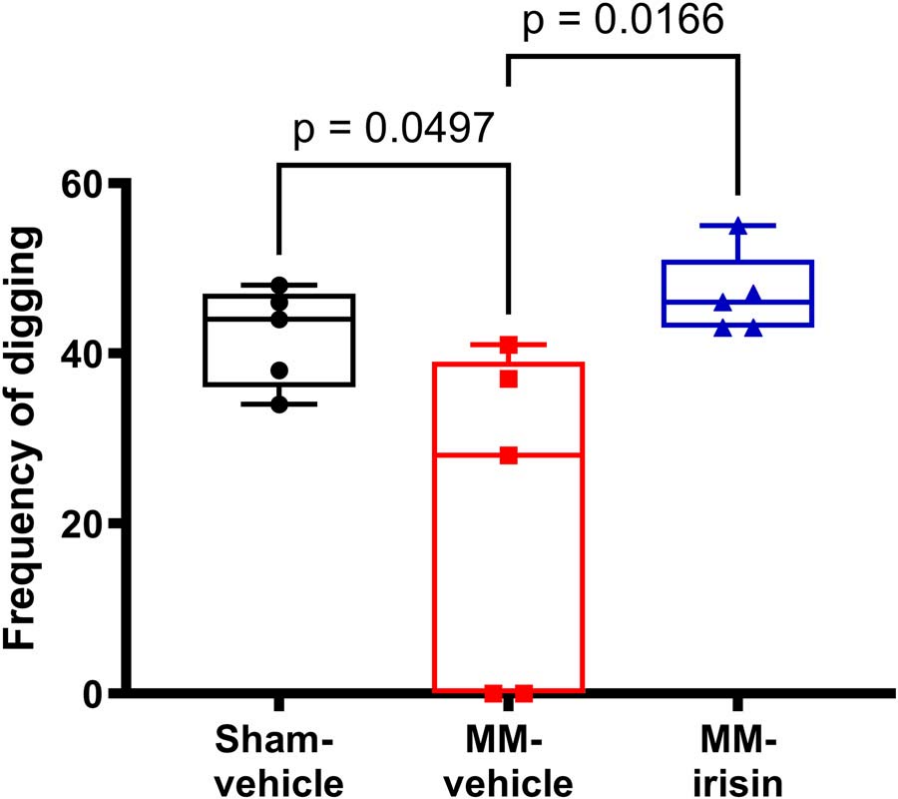
Irisin improves MM mice explorative abilities in the OFT. Evaluation of digging frequency in Sham-vehicle, MM-vehicle, and MM-irisin mice in the OFT. A one-way ANOVA with Tukey’s multiple comparisons tests was performed. Data are presented as box-and-whisker with median and interquartile ranges, from max to min, with all data points shown (*n* = 5 mice per group).

Despite no significant differences were observed between MM irisin and MM-vehicle mice regarding behavioral parameters such as total distance moved, velocity, and rearing (data not shown), the myokine significantly enhances the frequency of digging (*P* = .0166), which was significantly reduced in MM-vehicle group compared with sham-vehicle one (*P* = .0497, Figure 5), suggesting its possible activity in improving the explorative abilities in MM-mice.

### *Irisin reduces myeloma cell* in vitro *viability and modulates Notch 3 and Cyclin D1 gene expression*

**Figure 6:**
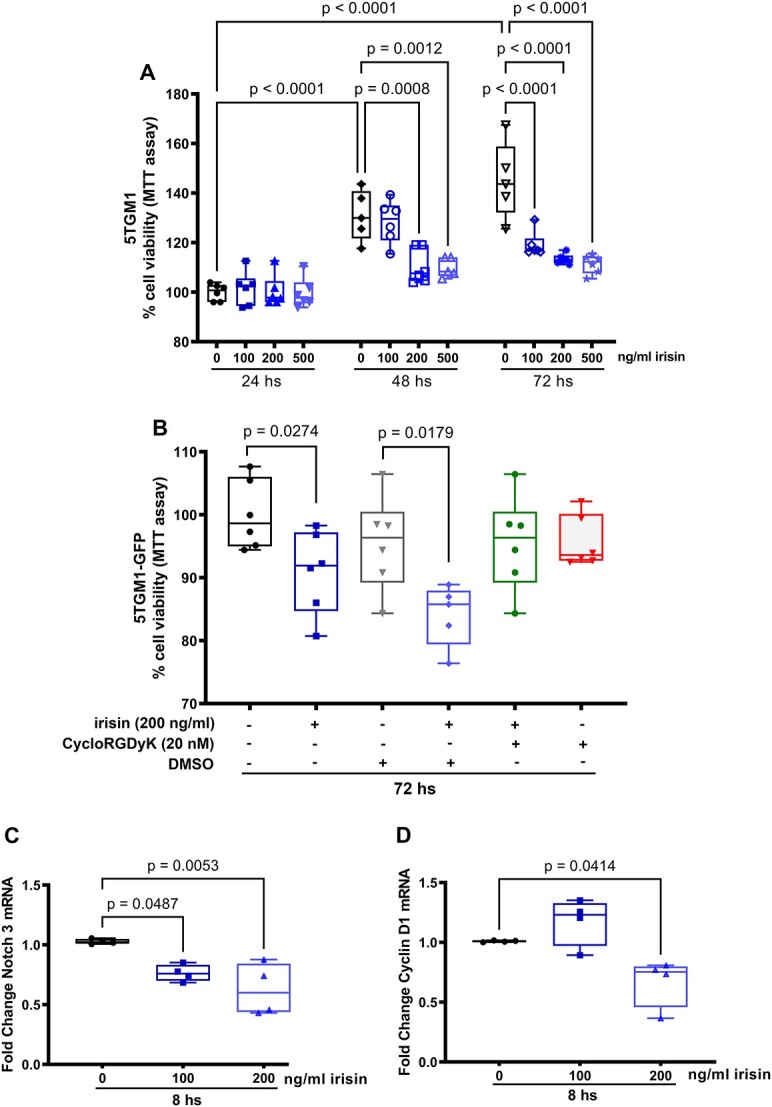
In vitro Irisin reduces 5TGM1 cell viability and Notch 3 and Cyclin D1 gene expression. MTT assay performed on 5TGM1 cells treated or not with different rec-irisin concentrations (100, 200, or 500 ng/mL) for different time periods (24, 48, or 72 h) (*n* = 6 for each group of treatment) (A). An ordinary one-way ANOVA with Tukey’s multiple comparisons tests was performed. Data are shown as a box-and-whisker plot with median and interquartile ranges, from max to min, with all data points shown. MTT assay performed on 5TGM1 cells pre-treated for 10 min with 20 nM of CycloRGDyK or DMSO, and then stimulated with 0 or 200 ng/mL rec-irisin for 72 h (*n* = 6 for each group of treatment) (B). Kruskal–Wallis test was performed. Data are shown as a box-and-whisker plot with median and interquartile ranges, from max to min, with all data points shown. Quantitative PCR (qPCR) showing modulation of mRNA expression levels of Notch 3 (C) and Cyclin D1 (D) and assayed on 5TGM1 cells treated or not (*n* = 4) with 100 ng/mL (*n* = 4) or 200 ng/mL (*n* = 4) of rec-irisin for 8 h. A one-way ANOVA with Tukey’s multiple comparisons tests was performed. Data are presented as box-and-whisker plots with median and interquartile ranges, from max to min, with all data points shown.

Based on in vivo reduction of myeloma cell invasion in the femur of irisin-treated MM mice, we studied in vitro effect of myokine on 5TGM1 cell viability by MTT assay. The cells were treated with rec-irisin at increasing concentrations (100 ng/mL, 200 ng/mL, or 500 ng/mL) for different times (24, 48, or 72 hs) (*n* = 6 for all groups). Interestingly, rec-irisin significantly reduces myeloma cell viability after 48 hs at both 200 ng/mL (*P* = .0008) and 500 ng/mL (*P* = .0012); after 72 hs, the cell viability decrease is detectable at the lowest irisin concentration (100 ng/mL, *P* < .0001), as well as at higher concentrations (*P* < .0001) (Figure 6A). To evaluate αV integrin involvement in mediating irisin effect on 5TGM1 cell viability reduction, we pre-treated these cells for 10 min with 20 nM of CycloRGDyK, an inhibitor of αVβ3 and αVβ5 integrins, or DMSO, the vehicle in which the inhibitor was dissolved, and then we stimulated the cells with 0 or 200 ng/mL rec-irisin for 72 hs. As shown in Figure 6B, irisin inhibitory effect was confirmed on MM cell viability (*P* = .0274) as also demonstrated in the presence of DMSO used as negative control (*P* = .0179). Indeed, CycloRGDyK pre-treatment abrogated irisin ability to reduce MM cell viability, suggesting a possible involvement of integrins in this effect.

Based on the importance of homotypic Notch 3 signaling and Cyclin D1 expression in the regulation of MM cell proliferation,^40^ we evaluated the expression levels of Notch 3 and Cyclin D1 on 5TGM1 cells treated or not (*n* = 4) for 8 hs with 100 ng/mL (*n* = 4) or 200 ng/mL (*n* = 4) of rec-irisin. We demonstrated a significant reduction in Notch 3 gene levels at both concentrations tested (*P* = .0487 and *P* = .0053, respectively, Figure 6C), while no effect was found on Notch 1, Notch 2, Notch 4, Jagged 1, and Jagged 2 (data not shown). We further found that stimulation with rec-irisin at 200 ng/mL significantly reduced Cyclin D1 gene levels (*P* = .0414, Figure 6D), compared to MM-untreated cells.

## Discussion

Bone disease is characterized by osteolytic lesions, and skeletal-related events (SREs), such as compression of the spinal cord and pathological fractures, which worsen daily activities, reduce quality of life, and increase patients’ risk of mortality.

Although current therapies have resulted in lengthened survival of patients, BD treatment still has several critical issues and remains an unmet priority. Indeed, in addition to current antiresorptive drugs, research now focuses on osteoanabolic molecules that, in combination/alternative to the existing ones, can help to better manage bone loss in MM.

The present study demonstrates that the new exerkine irisin, produced by skeletal muscle after physical activity, administered to MM female mice, exerts beneficial effects on MMBD by partially preventing trabecular bone damage and reduces the homing of malignant cells by reducing their viability, possibly through inhibition of Notch 3 and Cyclin D1.

The interest in exploring the effects of irisin on MMDB arose from our previous in vivo studies, performed in healthy young mice and in a mouse model of disuse-induced osteosarcopenia, which demonstrated, respectively, that irisin has a bone anabolic effect and prevents the onset of osteoporosis or rescues cortical and trabecular bone loss.^8^^,^^9^ Moreover, it was also demonstrated that irisin prevents trabecular bone loss in the femur and in the tibia of mice with estrogen deficiency-induced osteoporosis^41^ and protects against trabecular BMD decrease in the tibia of mice with androgen deficiency-induced osteoporosis.^13^

Intriguingly, in an MM-induced mouse model, we here demonstrated the ability of irisin to improve some bone parameters, affected by bone damage occurrence. Our μCT analysis revealed significant deterioration of femoral trabecular microarchitecture in MM mice partially regained in rec-irisin-treated ones. Intermittent intraperitoneal administration of the myokine for 5 wk partially prevents, in the femur, the reduction of femur BV/TV and trabecular number, the increase of trabecular separation, and the decline of the fractal dimension of the trabeculae detected in untreated MM mice. These results suggest that irisin contributes to decreased bone damage in MM through its ability to increase Opg gene levels in the femoral BM and reduce RankL protein expression and RankL/Opg ratio in the osteocyte-rich femoral bone. These results are in agreement with Delgado-Calle et al.,^32^ who reported that in mice, osteocytes interacting with MM cells, express higher RankL levels than those of control mice. The ability of irisin to modulate molecules involved in osteoclast formation or inhibition in favor of a reduction in bone resorption appears to be of great interest, as it has been established for many years that the imbalance of the RankL/Opg system in the local BM microenvironment is strongly responsible for the occurrence of MM bone lesions.^42-44^ It is well known that malignant plasma cells, which proliferate continuously in the BM, increase RankL and inhibit Opg production by stromal cells and osteoblast.^37^ In mice, Opg reduction leads to osteoporosis,^45^ and its administration inhibits osteoclast differentiation and increases tibial, femoral, and vertebral BMD, thus preventing the development of osteolysis.^46^ In humans, the RankL/Opg ratio is significantly higher in patients with MM than in healthy controls, it is associated with elevated bone resorption and osteolytic lesions,^47^ and its assessment could be a projection of MMBD severity.^48^ The importance of molecules involved in bone resorption in MMBD is such that in recent years an Opg-mimetic drug, Denosumab, has been developed, approved, and successfully used to prevent the onset of new osteolysis.^49^

Although irisin modulates molecules involved in the restoration of altered bone resorption in MM, we did not find any change in the circulating levels of the bone resorption marker, CTX, in the 3 groups of mice. This evidence is probably due to the restriction of the tumor burden to the BM of tibia and femur. However, in MM-irisin-treated mice, we found increased levels of the marker of bone formation, PINP, which levels decreased in MM-untreated mice. This finding is in line with our demonstration that irisin stimulation induces Sclerostin reduction in the femoral bone of MM mice, therefore contributing to the improvement of bone formation. It is important to note that our evidence about the reduction of Sclerostin protein levels in the cortical bone of MM mice is in agreement with literature data showing higher Sclerostin production by osteocytes in a mouse model of MM.^32^ Other authors also demonstrated that anti-Sclerostin antibody prevented MMBD and fracture risk in MM mice by increasing osteoblast numbers and bone formation.^50^ Delago Calle et al. also demonstrated that the administration of anti-Sclerostin antibody increased PINP serum levels and bone mass in MM mice.^33^ Thus, it can be possible that, in our MM model, through the irisin-induced Sclerostin reduction, the myokine increases osteoblast activity and, subsequently, enhances PINP circulating levels. This hypothesis is consistent with our previous work in which we showed that, in an osteoporotic-induced mouse model, intermittent irisin treatment identical to that performed in this work decreased bone Sclerostin levels compared with osteoporotic untreated mice.^9^ Of note, the dosage and administration strategy of irisin injection are particularly important for the effect elicited. Indeed, our findings, in contrast with those of Kim et al.^12^ showing that irisin increased Sost mRNA levels in cortical bone of mice, could depend on the different dose and administration strategies utilized. In particular, Kim et al. injected rec-irisin at a dosage 10 times higher than ours, given continuously daily for 6 d. The importance of Sclerostin in MM came also from human studies demonstrating that Sclerostin serum levels positively correlated with decreased osteoblast function, disease stage, and fractures.^38^^,^^39^ Differently from Sclerostin, protein levels of Dkk1, another important inhibitor of bone formation, did not change in the cortical bone of our mice groups. This could be due to a selective effect of the myokine, which targets Sclerostin and not other molecules involved in the inhibition of bone formation. Furthermore, the possibility that MM plasma cells can contribute to irisin-induced Sclerostin modulation needs to be further investigated. This is because Eda et al. data demonstrated that Dkk1 production by MM plasma cells stimulates Sclerostin expression in osteocytes and osteoblasts.^38^ Therefore, future studies may elucidate the possibility that in a mouse model of MM, Sclerostin inhibition by irisin may be mediated by the modulation of Dkk1 secretion from plasma cells.

Thus, our findings are overall consistent with literature data, as the partial prevention of trabecular bone damage induced by irisin in MM mice may be due to its ability to decrease both the RankL/Opg ratio and Sclerostin levels, shifting the balance of molecules influencing bone remodeling in favor of osteoprotective ones. In parallel, in our MM mouse model, irisin reduces tumor burden, as it negatively modulates, in the sera, IgG2b levels, a paraprotein detection commonly used to assess the onset of MM in mice and inhibits the homing of myeloma cells in the BM of femurs. These findings are in line to some extent with the effect of irisin on malignant plasma cell viability in vitro. Literature evidence has shown that irisin can modulate the proliferation of many cancer cells. In particular, in vitro studies demonstrated that irisin inhibited the number of malignant breast,^24^ prostate,^26^ lung,^27^ ovarian^25^ and pancreatic cancer cells^28^ as well as osteosarcoma cells,^23^ but the effects on myeloma cells have not been studied up to now. Sabol et al recently demonstrated that Notch 3 signaling, activated through homotypic plasma cell interaction, contributes to their growth by upregulating Cyclin D1.^40^ These authors demonstrated that murine 5TGM1 cells knocked down for Notch 3 expression showed both a 30%–35% time-dependent reduction in cell number compared with control MM cells and a 90% reduction in Cyclin D1mRNA expression. Based on this knowledge, our finding showing the ability of irisin to reduce the expression of Notch 3 and Cyclin D1 in MM cells suggests that the decrease in myeloma cell viability could occur through the inhibition of Notch 3 and Cyclin D1. Moreover, in agreement with Kim et al.,,^12^ our results show that the irisin ability to inhibit cell viability probably involves αV integrins, as their inhibition counteracts the effect of the myokine.

No less important, our data reveal that systemic administration of irisin impacts on mice locomotor activity by increasing the frequency of digging, an exploratory behavior related to self-protection, indicative of well-being. For the first time, we showed that this exercise-mimetic myokine can exert a positive effect on exploratory behavior in mice with MM, helping to improve decreased mobility in MM.

Thus, our study reveals exciting data on the role of irisin in MM as a potential future pharmacological strategy for ameliorating bone disease and reducing tumor growth although the involved mechanisms need to be better elucidated. Particularly, it remains unclear and represents a limitation of this work, what is the reason why in the inoculation site, the tibia, there is only a trend in tumor burden reduction, which instead becomes significant in the femur. Our findings are even more intriguing in the search for new anabolic agents in MMBD treatment as irisin seems to be a good candidate for improving BD by acting in two directions: on bone formation, through the decrease of Sclerostin, and on bone resorption by reducing RankL/Opg ratio.

Moreover, despite the need for further studies, irisin could influence the quality of life both for the beneficial effect on bones and for the improvement of locomotor activity in our mouse model of MM.

## Author contributions

Roberta Zerlotin (Conceptualization, Formal analysis, Investigation, Methodology, Resources, Writing—original draft, Writing—review & editing [equal]), Angela Oranger (Conceptualization, Formal analysis, Investigation, Methodology, Resources, Writing—original draft, Writing—review & editing [equal]), Patrizia Pignataro (Conceptualization, Methodology, Resources, Software, Validation), Manuela Dicarlo (Methodology, Software, Validation), Lorenzo Sanesi (Formal analysis, Validation), Clelia Suriano (Formal analysis, Investigation, Methodology, Validation), Giuseppina Storlino (Formal analysis, Investigation, Methodology), Rita Rizzi (Formal analysis, Validation), Anna Mestice (Formal analysis, Investigation), Sante Di Gioia (Methodology), Giorgio Mori (Formal Analysis, Software, Validation), Maria Grano (Conceptualization, Data curation, Funding acquisition, Project Administration, Resources, Supervision), Graziana Colaianni (Conceptualization, Data curation, Project administration, Software, Supervision, Validation, Writing—review & editing), and Silvia Colucci (Conceptualization, Data curation, Formal analysis, Investigation, Resources, Project administration, Supervision, Validation, Visualization, Writing—original draft, Writing—review & editing)

All authors have read and agreed to the published version of the manuscript.

## Funding

This research was supported by funding from Regione Puglia and CNR for Tecnopolo per la Medicina di Precisione. D.G.R. n. 2117 of 21.11.2018 (CUPB84I18000540002)—C.I.R.E.M.I.C. (Research Center of Excellence for Neurodegenerative Diseases and Brain Aging)—University of Bari “Aldo Moro,” and from Next Generation EU [DM 1557 11.10.2022], in the context of the National Recovery and Resilience Plan, Investment PE8—Project Age-It: “Ageing Well in an Ageing Society”(CUP: B83C22004800006 to M.G.). The opinions and views expressed are only those of the authors and do not necessarily correspond to those of the European Union or the European Commission, which are not responsible for them.

## Conflicts of interest

All the authors declare that there is no conflict of interest regarding the publication of this work. All authors read and approved the final version of the submitted manuscript and consent to be responsible for all aspects of the research. They ensure that all questions related to the integrity or accuracy of the research are properly explored and solved.

## Data availability

The data that support the findings of this study are available on request from the corresponding author.

## Supplementary Material

R2_Antibodies_Supplemental_Table_ziae066

R2_Primers_Supplemental_Table_ziae066
