# Supplementary figures and images for: Irisin prevents trabecular bone damage and tumor invasion in a mouse model of multiple myeloma
Source: JBMR Plus. 2024 May 23;8(7):ziae066. doi: 10.1093/jbmrpl/ziae066 (PMC11162589; doi:10.1093/jbmrpl/ziae066)

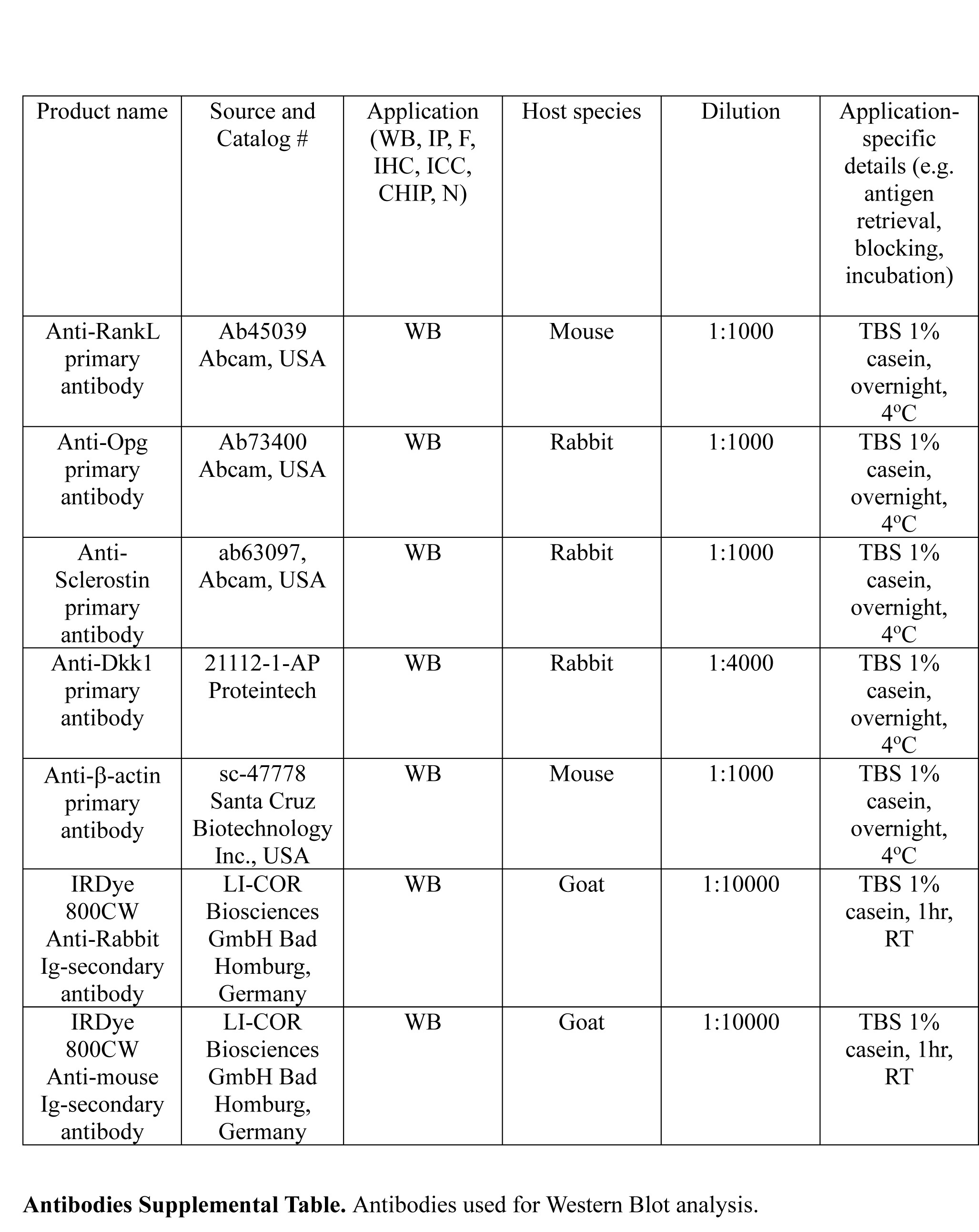

Supplement: R2_Antibodies_Supplemental_Table_ziae066 [file r2_antibodies_supplemental_table_ziae066.jpeg]

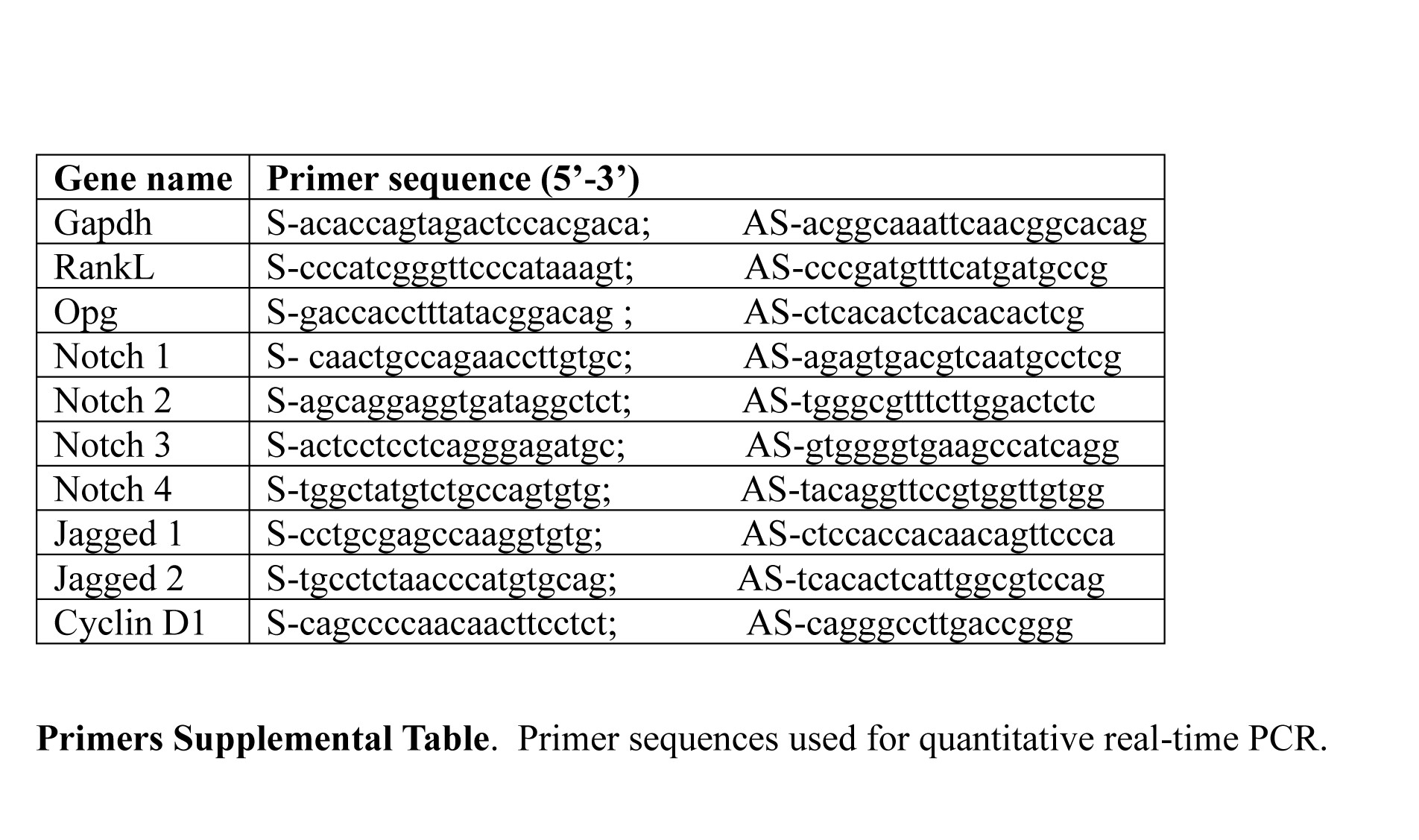

Supplement: R2_Primers_Supplemental_Table_ziae066 [file r2_primers_supplemental_table_ziae066.jpeg]
